# Complete Coding Genome Sequence for Mogiana Tick Virus, a Jingmenvirus Isolated from Ticks in Brazil

**DOI:** 10.1128/genomeA.00232-17

**Published:** 2017-05-04

**Authors:** Erika C. Villa, Sandra R. Maruyama, Isabel K. F. de Miranda-Santos, Gustavo Palacios, Jason T. Ladner

**Affiliations:** a Center for Genome Sciences, United States Army Medical Research Institute of Infectious Diseases, Fort Detrick, Maryland, USA; b Department of Genetics and Evolution, Federal University of São Carlos, São Carlos, São Paulo, Brazil; c Faculdade de Medicina de Ribeirão Preto, Universidade de São Paulo, Ribeirão Preto, São Paulo, Brazil

## Abstract

Mogiana tick virus (MGTV) is a segmented jingmenvirus isolated in 2011 from cattle ticks in Brazil. Here, we present a complete coding genome sequence for MGTV isolate MGTV/V4/11, including all four segments. MGTV is evolutionarily related to the Jingmen tick virus isolates SY84 and RC27.

## GENOME ANNOUNCEMENT

The jingmenviruses are a group of segmented, and likely multicomponent, viruses that are evolutionarily related to the unsegmented viruses of the genus *Flavivirus* (1). Although the jingmenvirus group has only recently been recognized, with the first virus descriptions published in 2014 (2, 3), known viruses in this group are diverse, globally distributed, and capable of infecting a wide range of animal hosts (1–5). Here, we report the complete coding genome sequence (i.e., only missing portions of terminal untranslated regions) (6) of the MGTV/V4/11 isolate from cattle ticks (*Rhipicephalus microplus*) collected from Holstein bulls in Ribeirão Preto, São Paulo state, Brazil. Mogiana tick virus (MGTV) was one of the earliest jingmenviruses to be reported, and at the time of publication, the segmented nature of the genome was not understood. Therefore, only the two genome segments with detectable sequence homologies to flaviviruses were originally reported (2). We revisited the data set of Maruyama et al. (2) and assembled the complete coding sequences for all four genome segments.

We downloaded the raw Illumina sequence reads from the NCBI Sequence Read Archive (GenBank accession numbers SRA055953/SRR525284), which includes 11,898,134 2 × 150-bp paired-end reads. We assembled the reads *de novo* using SPAdes version 3.7.1 (7) and identified MGTV genome segments through sequence similarity (BLASTN) to the published genome of Jingmen tick virus (JMTV) isolate SY84 (GenBank accession numbers KJ001579 to KJ001582). We then realigned the reads to the MGTV contigs using Bowtie 2 version 2.0.6 (8), recalled the consensus using SAMtools version 0.1.18 (9) along with custom scripts (https://github.com/jtladner/Scripts/blob/master/reference-based_assembly/consensus_fasta.py), and masked any positions with <3× coverage in support of the consensus. We used Cutadapt version 1.9.dev1 (10) to remove Illumina adaptors, Prinseq-lite version 0.20.3 (11) to trim and filter low-quality reads/bases, and Picard (http://broadinstitute.github.io/picard) to remove duplicates. We only used bases with Phred quality score ≥20 in consensus calling.

The *de novo* assembly resulted in 534,445 contigs, 4 contigs of which exhibited significant similarity to JMTV isolate SY84. Segments 1 and 3 were previously published as nonstructural protein 5 (GenBank accession no. JX390986.1) and nonstructural protein 3 (GenBank accession no. JX390985.1), respectively. We extended segment 1 by 573 nucleotides (nt) on the 5′ end and 543 nt on the 3′ end, and we extended segment 3 by 241 nt on the 5′ end and 60 nt on the 3′ end. We found no mismatches between our assemblies and those of Maruyama et al. (2). Segments 2 and 4 were previously unpublished. MGTV/V4/11 contains putative open reading frames congruent with JMTV NSP1 (segment [Seg] 1), NSP2 (Seg 3), VP1 (Seg 2), and VP2-3 (Seg 4) (3), and the genetic divergences between MGTV and JMTV SY84 and RC27 ranged from 9.7 to 12% at the nucleotide level and from 3.2 to 7.6% at the amino acid level. Given these similarities, MGTV may belong to the same species as JMTV.

### Accession number(s).

GenBank accession numbers JX390985 (segment 3) and JX390986 (segment 1) have been updated to complete coding segments. The new annotations have been deposited into GenBank as accession numbers KY523073 (segment 2) and KY523074 (segment 4).
